# Detection of a new insect flavivirus and isolation of *Aedes* flavivirus in Northern Italy

**DOI:** 10.1186/1756-3305-5-223

**Published:** 2012-10-08

**Authors:** David Roiz, Ana Vázquez, Fausta Rosso, Daniele Arnoldi, Matteo Girardi, Laureano Cuevas, Esperanza Perez-Pastrana, Mari Paz Sánchez-Seco, Antonio Tenorio, Annapaola Rizzoli

**Affiliations:** 1 Wetland Ecology Department, Doñana Biological Station, CSIC, Sevilla, Spain; 2 Biodiversity and Molecular Ecology Department, Research and Innovation Centre - Fondazione Edmund Mach. San Michelle all’Adige, Trento, Italy; 3 Laboratory of Arboviruses and Viral Imported Diseases. National Center of Microbiology. Institute of Health “Carlos III”, Majadahonda, Madrid, Spain; 4 Electron Microscopy Department, National Center of Microbiology. Institute of Health “Carlos III”, Majadahonda, Madrid, Spain

**Keywords:** *Aedes albopictus*, *Aedes cinereus/geminus*, Italy, *Aedes* flavivirus, Integrated sequences, Insect flavivirus, Non-retroviral integrated RNA viruses

## Abstract

**Background:**

During recent years, numerous novel ‘insect flaviviruses’ have been discovered in natural mosquito populations. In a previous study we described the presence of flavivirus DNA sequences integrated in *Aedes albopictus* (Asian tiger mosquito) populations from Northern Italy in 2007.

**Methods:**

During 2008 we collected and tested *Aedes* females for flavivirus presence and developed phylogenetic analysis, virus isolation, electron microscopy studies and RNAse treatments.

**Results:**

We detected a high prevalence of flavivirus in *Ae. albopictus* (77.5%). The phylogenetic analysis identified the insect flavivirus sequences as *Aedes* flavivirus (AEFV) recently described in Japan, and that may have been introduced in Italy travelling with the tiger mosquito. Some of these pools grew in C6/36 cells, producing cytopathic effects, and the RNase treatment results showed the presence of the detected sequences in RNA forms. Furthermore, we detected a new insect flavivirus in one pool of *Aedes cinereus/geminus* mosquitoes. Phylogenetic analysis of this virus shows that it forms a distinct cluster within the clade of insect flavivirus.

**Conclusions:**

This is the first study to report a high prevalence, to describe the seasonal activity and an isolation of the insect flavivirus *Aedes* flavivirus in Europe. Moreover we describe the detection of a new insect flavivirus detected from *Ae. cinereus* mosquitoes from Italy. These flavivirus may be common, ubiquitous and diverse in nature and we discuss the implications of the insect flavivirus group in virus evolution and transmission.

## Background

The increasing trend of global travel and trade are causing changes in the distribution of arboviruses worldwide. Arboviruses could be introduced by travellers, migratory birds or vectors carried through international trade. One paradigm of this effect of global change is the Asian tiger mosquito (*Aedes albopictus*), introduced in Italy by the used tire trade from Asia and the colonisation of a large part of the country [1]. *Ae. albopictus* has been involved in an outbreak of Chikungunya virus (CHIKV) (Togaviridae family, *Alphavirus* genus) in Emilia-Romagna by a traveller returning from India [2] and has been associated to endemic transmission of the Dengue virus (DENV) in France [3] and Croatia [4]. In this scenario, surveillance of arboviruses in field-collected mosquitoes is an important tool for detecting emerging viruses in Europe, including the genus *Flavivirus,* grouping many important human pathogens. There are about 70 known flaviviruses, and they are a heterogeneous group including species capable of infecting vertebrates and/or insects. Mosquito-borne viruses (MBV) and tick-borne viruses (TBV) are the most important groups due to their public health implications, highlighting the four serotypes of DENV, currently resurging in several areas of the world [5], tick-borne encephalitis virus (TBEV), yellow fever virus (YFV) and the Japanese encephalitis virus (JEV) [6]. Recently, the mosquito-borne flavivirus West Nile virus (WNV) has emerged in North America [7] and several parts of Europe, such as Italy [8] and Greece [9]. In Europe Usutu (USUV) and Bagaza viruses (BAGV) also recently appeared [10,11].

There are two other groups of flaviviruses: “Unknown arthropod vector” (UNKV) isolated from mammals, and ‘insect flaviviruses’ or ‘insect-specific flaviviruses’ [12,13]. The first virus characterised within insect flaviviruses group, was Cell fusing agent virus (CFAV), isolated from *Aedes aegypti* cells [14]. CFAV replicates in mosquitoes and in *Ae. albopictus* cells [15] but not in mammal cells, and the complete genomic sequence has been characterised [16]. CFAV has been isolated from natural populations in Puerto Rico [17], and Thailand [18]. To date, several other insect flaviviruses have been described. In 2003, Kamiti River virus (KRV) was isolated from field-collected *Aedes macintoshi* from Kenya [19]. Moreover, *Culex* flavivirus (CXFV) has been isolated and characterised from *Culex sp.* mosquitoes in Japan, Indonesia, Guatemala, Trinidad, United States, Mexico and Uganda [20-27]. Other new members of the insect flaviviruses group are Quang Binh Virus (QBV) isolated from *Cx. tritaeniorhynchus* in Vietnam [28]; Nakiwogo Virus (NAKV) isolated from *Mansonia africana nigerrima* in Uganda [22] and Calbertado virus detected mainly in *Cx. tarsalis* mosquitoes in North America [29]. *Aedes* flavivirus (AEFV) has been isolated from *Aedes albopictus* and *Aedes flavopictus* mosquitoes from Japan and is related to CFAV and KRV [30]. In Europe, several insect flaviviruses have been detected in *Ochlerotatus* sp.*, Culex* sp. and *Aedes* sp. from Italy, Spain, Portugal, United Kingdom and Czech Republic [31-35].

The aim of this study is to describe: i) the isolation of one strain of AEFV with a high prevalence in a population of *Ae. albopictus* in Northern Italy and ii) the detection of a new insect flavivirus discovered in *Aedes cinereus/geminus* mosquitoes in 2008.

## Methods

### Study area and field work

The study was conducted in the Province of Trento, in the municipalities of Arco and Riva del Garda (Trentino-Alto Adige, Northern Italy). These areas boast a mild, Mediterranean climate, due to its northerly position near Lake Garda. Some mosquito species detected in this area are *Ae. albopictus*[36], *Cx. pipiens* and *Cx. hortensis*[37]. Twenty collection sites were chosen for the field research. Adult mosquito collections were made by BG-sentinel traps (BioGents, Regensbourg, Germany), which are, compared with other traps [38], very efficient in capturing adult *Ae. albopictus* and *Ae. aegypti.* BG-traps were located in suitable places for mosquitoes in private gardens, garden centers and school playgrounds, and were connected to an electricity supply. Each trap had a BG-lure attractant. During all the sampling period, every 48 hours, mosquitoes were removed by replacing the catch bag with another one, transported to the laboratory and killed at −20°C. The use of these traps ensured the survival of the adult mosquitoes until they arrived to the laboratory, preventing RNA degradation by the metabolic system of the insect. Field work was carried out from May to November 2008. The influence of climatic changes on *Ae. albopictus* numbers, activity and distribution and the potential effect of climate change have been described [39,40].

### Identification and storage

Mosquitoes were placed on a Petri dish with white filter paper on a chill table and the species were identified using a stereomicroscope and the appropriate keys [41]. It was not possible to identify individuals belonging to the sibling mosquito species *Aedes cinereus* and *Ae. geminus*, genera *Aedes,* subgenera *Aedes,* group *cinereus* and they were named as *Aedes cinereus/geminus*[42,43]. Mosquitoes consisting of 1 to 56 mosquitoes were pooled by species, sex, localities and date. To these pools, if more than 30 mosquitoes were present, 700 μl of MEM (Minimum Essential Medium Eagle, Invitrogen) was added, and if there were less than 30 mosquitoes, 500 μl of MEM was added. Mosquitoes were triturated in MEM, and the homogenate was centrifuged at 13.000 rpm for 5 min at 4°C. 140 μl of the supernatant was collected and dissolved in 560 μl of AVL carrier/buffer RNA solution (Qiagen). This lysis buffer renders RNA viruses in samples inactive and stabilizes viral RNA before extraction [44]. Homogenates were prepared with sterile, RNAase-free pestles and 1.5 ml tubes. All the samples were stored at −80°C.

### Virus identification by RT-PCR, sequencing and phylogenetic analysis

Viral RNA was extracted from mosquito homogenates by using the QIAamp® Viral RNA Mini Kit (QIAGEN) according to the manufacturer’s instructions. Flavivirus detection was performed using a generic nested RT-PCR designed in the NS5 gene [45]. A modification was done in the nested PCR, adding the HotMaster Polymerse (Promega) to eliminate unspecific bands. Positive samples were sequenced in both directions and the obtained sequences were compared with those available in public databases.

With the aim of clarifying the phylogenetic relationships of the detected virus, and obtaining a phylogenetic study of the sequences, a generic RT-nested-PCR was used to amplify a major fragment of the NS5 gene [46] that generated an amplicon with enough phylogenetic information for proper taxonomic studies. The amplicons were purified by using the QIAquick® PCR Purification Kit (Qiagen) according to manufacturer’s instructions. Amplified cDNA was sequenced in both directions by using the ABI PRISM® BigDye® Terminator v3.1 Cycle Sequencing Kit. Sequences were obtained in SeqMan (DNAStar) and aligned using the program Mega v4.0 (Tamura et al., 2007) by using the ClustalW method [47]. Phylogenetic and molecular evolutionary analyses were conducted by using the program Mega v4.0 and the phylogenetic tree was built with the neighbour-joining (NJ) method, distance-p model and 1000 replications of bootstrap values. The sequences obtained in this study were compared with those available in public databases (GenBank), and representative insect flavivirus sequences were used in the phylogenetic analysis. Infection rates were calculated by biased maximum likelihood estimation rates (MLE) using the Poolscreen2 program [48], which calculates the prevalence of infection from the number of positive PCR pools using MLE. Graphics were done using Sigma Plot 9.0 (SPSS, Chicago, IL).

### Study for the presence of virus-specific DNA

In order to verify if positive pools were the result of RNA amplification and not DNA forms, 5 μl of nucleic acid extracts were digested with RNAsa A (Sigma) and incubated for 2 h at 37°C before amplification [49] and these treated extracts were directly amplified without a previous retro-transcription step. In a parallel analysis, each of these positive aliquots were assayed by RT-nested-PCR for flavivirus using 5 μl of untreated extracts to confirm that RNA was not degraded in the original pools.

### Virus isolation in C6/36 cells and electron microscopy studies

Virus isolation was attempted in C6/36 cell cultures, which were incubated at 33°C and the mosquito homogenates collected were used as inoculum. Cytopathic effects (CPE) were checked daily, and the culture supernatants were tested by RT-PCR. Culture supernatants were collected after a minimum of, three blind passages and stored as viral stocks at −80°C until tested by RT-PCR. The studies by electron microscopy were achieved in both fresh supernatants and cells from CPE positive cultures. The supernatants were fixed at a final concentration of 2% glutaraldehyde, clarified by low-speed centrifugation, ultracentrifugated at 35.000 rpm for 60 min in a Ty 50 Ti Beckman rotor at 4°C, and negative stained with PTA (phosphotungstic acid). The cells monolayers were fixed with 2% glutaraldehyde, put together with the cell pellets from the supernatant clarifications, dehydrated in serial ethanols, and embedded in epoxydic resin for ultrathin sectioning. The viral particles were identified, by both negative staining and ultramicrotomy, based on their ultrastructural characteristics in a Tecnai 12 or a Philips CM12 electron microscope at 120 kV [34].

## Results and discussion

**Figure 1:**
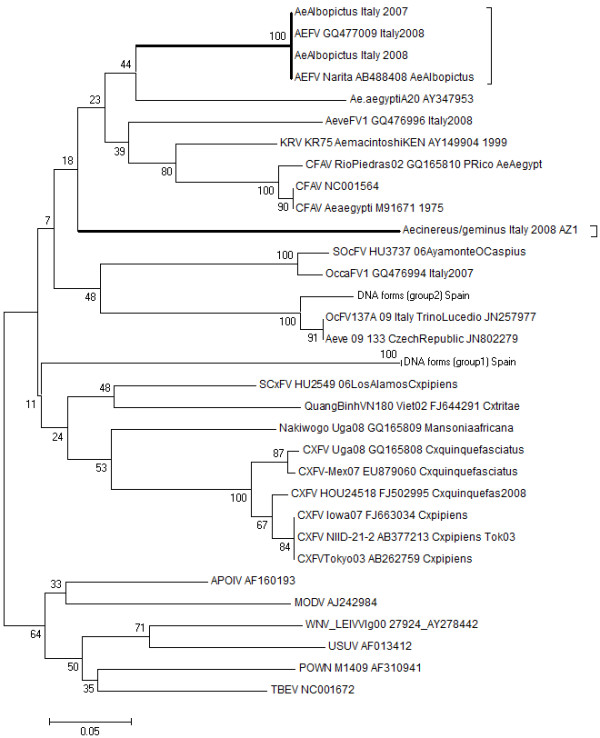
**Phylogenetic tree of the positive sequences based on the 84 nucleotides of the NS5 gene.** The tree was displayed by using the program Mega 5.05, Neighbor-Joining method and distance-p model with 1000 bootstrap replicates. The branches for flavivirus sequences published in the current study (AeAlbopictus Italy 2008 and *Aedes cinereus/geminus* Italy 2008 AZ1) are in bold. GenBank accession numbers for the insect flaviviruses sequences are indicated in the tree MBV: Mosquito-borne virus, TBV: tick-borne virus, UNKV: Unknown vector virus, AEFV: Aedes flavivirus, KRV; Kamiti River virus, CXFV: Culex flavivirus, CFA: Cell fusing agent.

In total, 129 pools (5029 individuals) of *Ae. albopictus* females and one pool (one individual) of *Ae. cinereus* were analysed; 107 (77.5%) were positive for flavivirus. The phylogenetic analysis performed in a short fragment of the NS5 gene (84 nucleotides), demonstrated that the sequences detected in this work were grouped in two clusters belonging to the insect flavivirus group (Figure 1). One cluster is formed by sequences obtained from *Ae. albopictus* mosquitoes, which are clustered with sequences from *Aedes* flavivirus (AEFV), the other cluster corresponds to a new sequence obtained from one pool of *Ae. cinereus*, which could belong to a new insect flavivirus. To ensure that positive pools were the result of RNA amplification, a treatment with RNAsa A was conducted, and the results showed that the sequences detected correspond to RNA forms.

**Figure 2:**
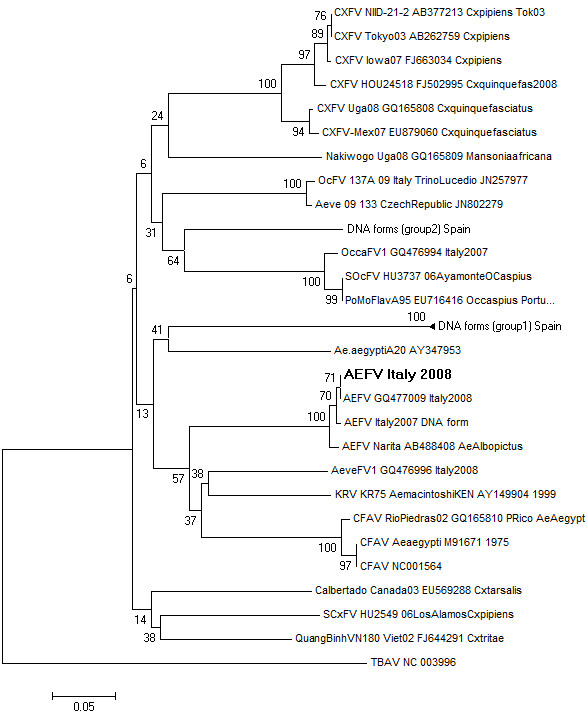
**Phylogenetic tree of the positive sequences based on 243 nucleotides of partial NS5 gene.** The tree was displayed by using the program Mega 5.05, Neighbor-Joining method and distance-p model with 1000 bootstrap replicates. The consensus flavivirus sequence obtained in the current study (AEFV Italy 2008) is in bold in the tree. The GenBank accession numbers for the AEFV sequences obtained in this work are **JX860634, JX860635, JX860636** and **JX860637.** AEFV: Aedes flavivirus, KRV: Kamiti River virus, CXFV: Culex flavivirus, CFA: Cell fusing agent and TABV: Tamana bat virus as outgroup.

**Figure 3:**
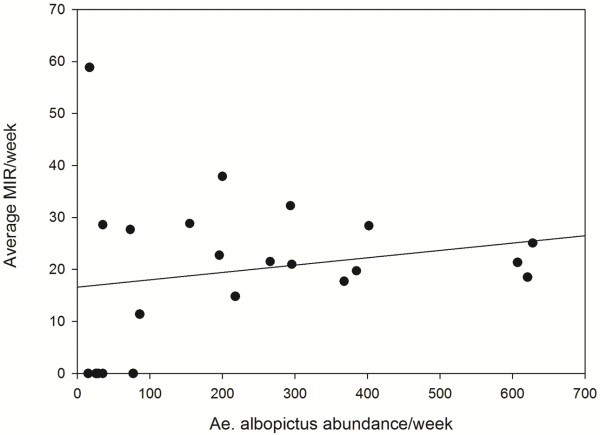
**Relationship between MIR (Minimum infection rate) and *Ae. albopictus *females abundance per week.**

**Figure 4:**
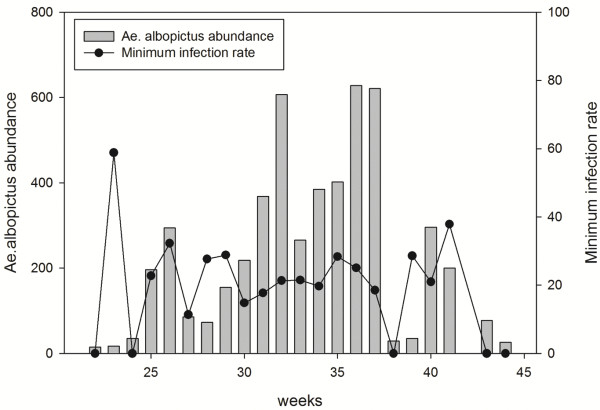
**Seasonal dynamics of *Ae. albopictus *female abundance and MIR (Minimum Infection rate) during the study period.**

The consensus sequence obtained from AEFV in a fragment of 84 nt of the NS5 gene, showed an identity of 100% with the DNA form sequence obtained from *Ae. albopictus* captured in the same area in 2007 [31]. And this sequence shared a 99-100% with the sequences of AEFV described in Japan [30] and Italy [50]. For this group of sequences, a final fragment of 969 nt of the NS5 gene [34] was obtained. However, the phylogenetic analysis was completed using 243 nt because this is the maximum length of the fragment available in GenBank for the majority of AEFV sequences. The phylogenetic tree showed that these sequences grouped (with robustness bootstrap values) with AEFV, described in Japan and Italy recently sharing 99% identity [30,50] and the integrated sequence detected in *Ae. albopictus* mosquitoes captured in Italy in 2007 in the same region [31] (Figure 2). Differences among localities for positivity were observed, with several collection sites (8/20) with all positive pools and one collection site with all negative pools. Therefore, the positive pools frequencies for AEFV vary between 0-100%, with a mean of 86.6% per locality. The MIR-MLE of the samples varies among localities with a mean from 0 to 54 and an upper limit of 154. There is a relationship between *Ae. albopictus* female abundance and the MIR for AEFV (Figure 3). Interestingly, observing the seasonal trend for mosquito abundance and MIR there is a peak of MIR in relation to mosquito abundance at the beginning of the season (Figure 4).

**Figure 5:**
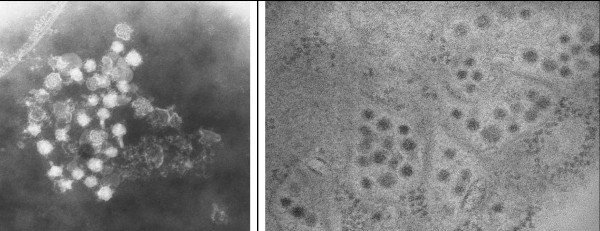
**Electron micrographs of C6/36 cells infected with AEFV.A**) PTA-Negative Staining of whole Flavivirus-like particles in culture supernatant. **B**) Ultramicrotomy. Thin section of infected cells: Flavivirus-like particles in cytoplasmic vesicles.

The AEFV detected in this work has been isolated in C6/36 cells and in the infected cultures, moderate or weak CPE were observed from 5–7 days post-infection. In these cell cultures with CPE, flavivirus-like particles were seen by transmission electron microscopy (EM) in both infected cells and the supernatant of the cell culture (Figure 5). The enveloped virions were approximately 50–60 nm in diameter. PCR assays performed on the supernatant of these cultures were conducted, and PCR-amplified products were obtained. We assume that this RNA detection was due to AEFV infection rather than residual RNA from the inoculum, because we could observe CPE, virus particles by EM and RNA in the cell culture of three consecutive serial passages. The nucleotide sequences have been submitted to the GenBank data bank (Accession numbers: BankIt1569634 Seq1 JX860634, BankIt1569655 Seq2 JX860635, BankIt1569658 Seq3 JX860636, BankIt1569661 Seq4 JX860637).

The new flavivirus sequence was detected in one pool of *Aedes cinereus/geminus* (small woodland mosquito) and grouped in a new cluster in the insect flavivirus group (Figure 1). This new sequence may correspond to a new insect flavivirus, henceforth named “*Aedes cinereus/geminus* flavivirus”, which shares 29% identity with CFAV, 33% with KRV, 34.5% with AEFV and 40% with CXFV in a short fragment of 84 nt. The nucleotide sequence is: CCCACAGTCGCCGGGGAGCCCAAAGGATCACGCACCATCTGGTACATGTGGTTGGGGTATTCGGTATCTCAATATGAGGCTCTC. This sequence amplified corresponds to RNA form, and this sample could neither be amplified using the NS5 amplification method nor could it be isolated in C6/36 cells. We compared the sequence obtained in this work with the sequence detected in 2010 from *Ae. cinereus* in the United Kingdom (sequence kindly provided by Claire Jeffries, Wildlife Animal Health and Veterinary Laboratories Agency, Surrey, UK) [35]. Only 31 nucleotides could be compared, and in this fragment both sequences shared a 94% of similarity. More sequence information is necessary for either samples to confirm that they are or not the same virus.

Currently, members of the insect flavivirus clade are considered to be viruses characteristic of mosquitoes, that appear to replicate only in mosquito cells and not in mammalian cells [12]. In recent years many tentative members of the insect flaviviruses group have been described worldwide. In this study, we present the detection and isolation of the insect flavivirus AEFV in *Aedes* mosquitoes from Northern Italy. Moreover we report the detection in *Aedes cinereus/geminus* mosquitoes from Italy of a sequence very similar to *Ae. cinereus* flavivirus detected in the same species in UK in 2010 [35].

In a previous study in the Trentino and Padova provinces, AEFV was detected in 2007, integrated into the genome of *Ae. albopictus* mosquitoes [31], already described in *Aedes* mosquitoes in Japan [30] and detected again in other areas of Italy [35,50]. In this work, an entomological survey was carried out in the same area one year later and AEFV was isolated. We have detected the same sequence of AEFV now, but in RNA form. The results of this survey demonstrated that the AEFV detected has a high prevalence (mean of 86.6%) in the *Ae. albopictus* mosquito populations during the whole season. Its infection rate increases with mosquito abundance, and there is a peak of infection rate at the beginning of the season. The cause for this peak could be related to the survival of eggs during diapause through vertical transmission and the increase of the first generation of mosquito after the hatching from diapausing eggs. On the other hand, the increase of infection rate according to mosquito abundance may be related to horizontal transmission by venereal transmission.

The infection by insect flaviviruses may affect infection, replication, and propagation of mosquito-borne flaviviruses both in vivo and in vitro, and may have already produced negative or positive selective pressure on virus susceptibilities and/or vector competence of mosquitoes. In a study in Mexico a high prevalence of CXFV was observed in *Cx. quinquefasciatus,* suggesting that mosquitoes infected with this virus could be refractory or less susceptible to subsequent infection with WNV or other viruses (hypothesis of super-infection exclusion) [25]. Alternatively, the enhancement of WNV transmission in mosquitoes inoculated simultaneously with CXFV strain Izabal has been reported [51] and this positive ecological association has been also described in field-collected mosquitoes from Chicago (United States) co-infected with CXFV and WNV [27]. Moreover in an entomological survey carried out in Italy in 2009 two *Ae. albopictus* pools sampled in the same site and week were found positive for AEFV and USUV respectively. Simultaneous circulation of WNV and USUV in the survey area was highlighted by the occurrence of mosquito WNV- and USUV-positive pools, thus testifying a co-circulation of both viruses [52]. Therefore, more studies might be developed to clarify if the presence of this high prevalence of AEFV in the populations of *Ae. albopictus* in northern Italy could influence the vectorial capacity of other human pathogenic flaviviruses circulating in Europe, such as WNV or USUV.

If these insect flaviviruses are able to develop an immune response in the host, when they enter in contact with them during the blood feeding, is unknown at the present time. Moreover it would be very interesting to know if the possible immunity developed by the host against the insect flaviviruses, would be able to interfere with the immunity that the possible hosts, including animals or humans, have against pathogenic flaviviruses.

The phylogenetic analysis based on the NS5 gene of the sequences obtained in this study shows that these sequences fall into two different clusters within the insect flaviviruses which are more related to *Aedes-*associated flaviviruses, such as CFA, KRV and AEFV. One of these clusters corresponded to sequences of AEFV, and interestingly, the NS5 sequences of the AEFV isolated in this work, were similar to the original Asian AEFV. Therefore, our hypothesis about the common presence of AEFV in *Ae. albopictus* Italian populations is that the virus travelled inside the tire-travelling tiger mosquito from Asia to USA, and from USA to Europe [53,54]. The virus might survive in the mosquito population due to several mechanisms such as vertical and/or sexual transmission [55].

The other cluster corresponded to the sequence data detected in *Aedes cinereus/geminus* mosquitoes. This sequence is very similar to the sequence of *Ae. cinereus* flavivirus detected in the UK in 2010, but longer sequences are needed in order to make a comparison. It has been suggested recently that the group of *Aedes*-borne flavivirus is more diverse and widespread in nature than other-specific flavivirus [12]. These findings corroborate the hypothesis of multiple introductions in the insect flavivirus group, as CXFV appears to have been introduced numerous times to the New World, confirming a mechanism of dispersion of insect flavivirus inside the mosquito populations.

## Conclusions

This is the first study to report a high prevalence, the seasonal activity and the isolation of the insect flavivirus AEFV in Europe. Moreover we describe the detection of a new insect flavivirus detected from *Aedes cinereus/geminus* mosquitoes from Italy. These results, together with the number and diversity of sequences related to insect flaviviruses reported in recent years [20-25,29], suggest that this group of viruses may be common, ubiquitous and diverse in nature and that they are undersampled at present. Therefore, our understanding of the implications of the insect flavivirus group in virus evolution and transmission is currently limited and future research may lighten the significance of this group. Studies are on-going in order to achieve viral isolation of the new insect flavivirus described in this work, and to follow up its genetic characterization and phylogenetic analysis.

## Competing interests

The authors declare that they have no competing interests.

## Authors’ contributions

DR and AV design the study, DR and DA participated in field sampling, DR, FR, and MG developed the flavivirus detection, AV, DR and MPSS developed the phylogenetic analysis, virus isolation and DNA integration essays. LC, EP and AV developed the electron microscopy essays. DR, AV, AT and AR analysed the results. All the authors have been involved in drafting the manuscript and have read and approved the final version.

## Authors’ information

David Roiz is a postdoctoral researcher on mosquito-borne disease ecology. He works in several topics, including flavivirus, global change, eco-epidemiology, remote sensing, blood-feeding behaviour, control strategies, invasive species and others.

Ana Vázquez is a virology postdoctoral researcher on arboviruses. She works in several topics, mainly in flaviviruses like WNV, SLEV and DENV and other mosquito-borne diseases.
